# SOX2-high cancer cells exhibit an aggressive phenotype, with increases in stemness, proliferation and invasion, as well as higher metabolic activity and ATP production

**DOI:** 10.18632/aging.204452

**Published:** 2022-12-22

**Authors:** Marta Mauro-Lizcano, Federica Sotgia, Michael P. Lisanti

**Affiliations:** 1 Translational Medicine, School of Science, Engineering and Environment (SEE), University of Salford, Greater Manchester, United Kingdom

**Keywords:** cancer stem cells (CSCs), SOX2, FACS sorting, CSC isolation, stemness

## Abstract

Cancer stem cells (CSCs) are responsible for cancer recurrence, treatment failure and metastatic dissemination. As such, the elimination of CSCs represents one of the most important approaches for the future of cancer treatment. Among other properties, CSCs show the activation of particular cell signalling pathways and the over-expression of certain transcription factors, such as SOX2. Herein, we describe a new model system to isolate stem-like cancer cells, based on the functional transcriptional activity of SOX2. Briefly, we employed a SOX2-enhancer-GFP-reporter system to isolate cancer cells with high SOX2 transcriptional activity by FACS sorting. The over-expression of SOX2 in this sub-population was validated by Western blot analysis and flow cytometry. SOX2-high cancer cells showed CSCs features, such as greater mammosphere forming ability, validating that this sub-population was enriched in CSCs. To further explore the model, we analysed other stemness characteristics in MCF7 and MDA-MB-231 breast cancer cell lines, corroborating that SOX2-high cells were more metabolically active, proliferative, migratory, invasive, and drug-resistant. SOX2-high MDA-MB-231 cells also showed a loss of E-cadherin expression, and increased Vimentin expression, consistent with an epithelial-mesenchymal transition (EMT). Therefore, endogenous SOX2 transcriptional activity and protein levels are mechanistically linked to aggressive phenotypic behaviours and energy production in CSCs.

## INTRODUCTION

Several studies have highlighted the involvement of cancer stem cells (CSCs) in the pathogenesis of tumour recurrence, distant metastasis, and therapy-resistance. CSCs are a rare sub-population of tumour cells, with stem cell-like properties, characterized by an epithelial-mesenchymal transition (EMT), self-renewal, tumour-initiation capability, high proliferation rates and/or drug-resistance. They also show other characteristic properties, such as the i) expression of cell surface markers, such as CD44 and CD133, ii) high activity of the enzyme aldehyde dehydrogenase (ALDH), iii) and/or the dysregulation of the self-renewal signalling pathways, such as Wnt, Notch or Hedgehog [1]. In addition, the biological activities of CSCs are regulated by several pluripotent transcriptional factors, such as SOX2, NANOG, OCT4, KLF4, and MYC. In fact, somatic cells can be reprogrammed to become induced pluripotent stem cells (iPS), by ectopic over-expression of some of these transcriptional factors [2].

Considering the important role that CSCs play in tumour relapse and therapy resistance, the identification and isolation of CSCs becomes an essential experimental step for identifying additional hallmarks of cancer. In this regard, the isolation of CSCs has largely been based on the use of specific cell surface protein biomarkers [3]. Here, we used a different approach, based on recombinant expression of eGFP, under the transcriptional control of the regulatory elements of several different transcription factors.

SRY-box 2 (SOX2) belongs to the SOX family of high-mobility group transcriptional factors. SOX2 plays an essential role in embryonic development and in maintaining the stemness of embryonic cells and various adult stem cell populations. SOX2 expression and activity is dys-regulated in different cancer types. Moreover, there is evidence that SOX2 mediates resistance towards established cancer therapies, and it is thought to be over-expressed in CSCs, where is involved in the regulation of the stemness and self-renewal [4].

NANOG, a differentiated homeobox domain protein, is involved in propagation of embryonic stem cells and helps maintain their self-renewal properties and regulates the functions of multi-potent transcription factors. Abnormal expression of NANOG has been reported in human cancers, such as breast cancer, cervical cancer, or brain cancer, suggesting the implication of Nanog in tumourigenesis and cancer progression [5]. Several studies also indicate that Nanog plays an important role in regulating the self-renewal and proliferation of CSCs [6, 7].

Lymphoid enhancer-binding factor 1 (LEF1), a member of the T-cell factor (TCF)/LEF1 family of high mobility group transcription factor, is a downstream mediator of the Wnt-signalling pathway. Over-expression of LEF1 is associated with tumour progression and poor prognosis [8]. Various studies demonstrate the role of LEF1 in the maintenance of the self-renewal properties of CSCs as well as in cell migration, invasion, and the epithelial mesenchymal transition (EMT) [9].

In the present study, we have used a SOX2-enhancer-GFP-reporter system, as it proved to be a good model during our screening of various stemness-related transcriptional factors, to identify and purify sub-population(s) of MCF7 and MDA-MB-231 breast cancer cells, with low and high levels of SOX2-transcriptional activity, by flow cytometry analysis.

These SOX2-high cell sub-populations showed characteristic CSC features, such as greater efficiency in 3D mammosphere formation, as directly compared with than the SOX2-low MCF7 cells, consistent with the idea that this sub-population of cells was indeed enriched in CSCs.

## RESULTS

### Exploiting stemness pathways and GFP-reporter systems to enrich for CSC populations

Here, we aimed to assess a series of transcriptional reporter systems for the enrichment of cancer stem cells (CSCs). More specifically, we enriched MCF7 and MDA-MB-231 sub-populations of CSCs, based on the differential transcriptional activity of three distinct transcription factors all involved in stemness and self-renewal, namely SOX2, TCF/LEF1 and NANOG. For this purpose, their high transcriptional activity was linked to the recombinant expression of eGFP, allowing the detection of different cell sub-populations by flow cytometry.

Briefly, MCF7 and MDA-MB-231 cells were stably-transduced with a lentiviral construct, driving eGFP protein expression, under the control of the different transcription factor recognition elements. These DNA constructs also contained a puromycin-resistance cassette for antibiotic resistance selection. After MCF7 and MDA-MB-231 cancer cell lines were stably-transduced, they were subjected to flow cytometry to isolate the 5% highest GFP (GFP-high) and the 5% lowest GFP (GFP-low) sub-populations. In this manner, the GFP-high cells represent the SOX2-high, TCF/LEF1-high or Nanog-high cell sub-populations and are potentially a more stem-like population. On the contrary, the GFP-low cells represent a non-stem-like population, which serves as an internal control and reference point for phenotypic comparison.

**Figure 1:**
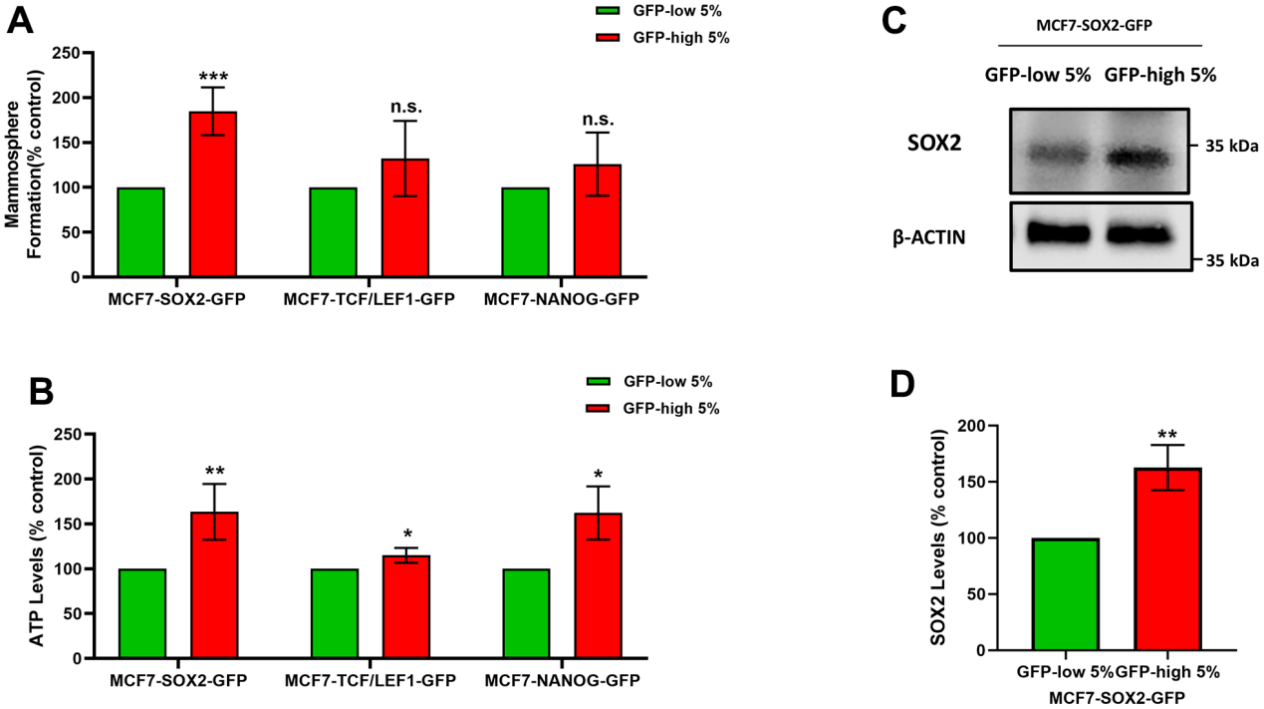
**SOX2-high MCF7 cells show an increased capacity for mammosphere formation and ATP production, with increased SOX2 protein expression levels.** MCF7 cells were stably-transduced with three different stem-cell related eGFP reporter systems (SOX2-GFP, TCF/LEF1-GFP and NANOG-GFP), that measure the transcriptional activity of these nuclear factors. Then, these different cell lines were subjected to cell sorting by flow cytometry to isolate the 5% highest GFP (GFP-high) and the 5% lowest GFP (GFP-low) sub-populations. (**A**) The GFP-high and GFP-low sub-populations were seeded in low-attachment plates for mammosphere assays and analysed after 5 days. (**B**) The GFP-high and GFP-low subpopulations were plated in complete DMEM medium and incubate with the Cell-Titer-Glo 2.0 Reagent for 15 min to determinate the ATP levels. (**C**) SOX2 levels of GFP-high and GFP-low subpopulations were assessed by Western blotting. β-actin was used as a protein loading control. (**D**) SOX2 levels of GFP-high and GFP-low subpopulations were analysed by flow cytometry. Note that the use of MCF7 cells harbouring the SOX2-GFP reporter system allowed for the enrichment of a GFP-high sub-population of cells, with increased mammosphere formation, higher ATP levels, and elevated expression of endogenous SOX2, consistent with an increase in SOX2 functional transcriptional activity. Experiments were performed at least 3 times independently. Results are shown as the mean ± SD: *t*-test, two tailed-test, *p < 0.05, ** p < 0.01, ***p < 0.001, n.s. not statistically significant.

Figure 1A shows that SOX2-high, TCF/LEF1-high and NANOG-high MCF7 cells all form mammosphere(s), with a greater efficiency as compared with the GFP-low cells. However, these differences were only statistically significant and higher in the SOX2-high cells, as compared to the SOX2-low cells. In contrast, the two other transcription factors, did not show significant differences upon further analysis.

Furthermore, the ATP levels (Figure 1B) are significantly increased in the GFP-high cells, as compared with the GFP-low cells in all cell lines, but the differences were greater in the SOX2-high cells, with respect to the SOX-low cells. Considering these results, we decided to focus on the use of the SOX2 transcriptional reporter system, as the best model system.

Finally, we also validated the expression of endogenous SOX2 in this cell model, after flow cytometry. More specifically, we show that GFP-high cells show higher levels of SOX2 endogenous protein expression by immunoblot analysis, as directly compared with GFP-low cells (Figure 1C). We also corroborated the higher levels of SOX2 by flow cytometry in GFP-high cells, as compared with their GFP-low counterparts (Figure 1D).

### SOX2-high MCF7 cells are more metabolically active and show an increase in CSC markers, mitochondrial mass, and cell proliferation

**Figure 2:**
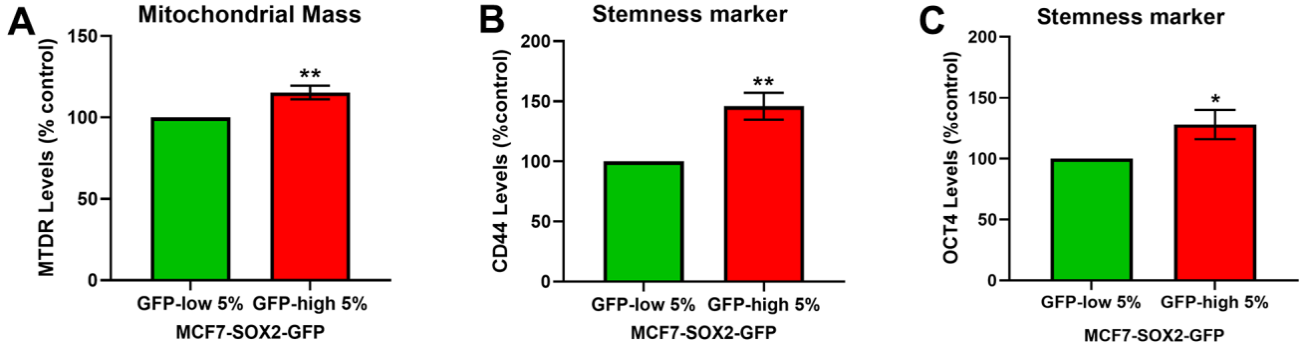
**SOX2-high MCF7 cells show a significant increase in mitochondrial mass and in stemness markers.** MCF7 cells stably-transduced with the SOX2-GFP reporter construct were subjected to FACS sorting to isolate the 5% highest GFP (GFP-high) and the 5% lowest GFP (GFP-low) subpopulations. (**A**) Mitochondrial mass was assessed with MitoTracker Deep-Red by flow cytometry. (**B**) CD44 levels were determined with an APC mouse anti-Human CD44 antibody by flow cytometry. (**C**) OCT4 levels were evaluated with a OCT4-PE antibody by flow cytometry. Experiments were performed at least 3 times independently. Results are shown as the mean ± SD: *t*-test, two tailed-test, *p < 0.05, ** p < 0.01.

To further validate the phenotype of SOX2-high MCF7 cells, we used a well-established fluorescent probe to quantitate mitochondrial mass, namely MitoTracker Deep-Red (Figure 2A), and two stemness markers, CD44 (Figure 2B) and OCT4 (Figure 2C), by FACs analysis. FACS quantitation of median fluorescence intensity (MFI) shows that both measures are significantly elevated in SOX2-high MCF7 cells.

**Figure 3:**
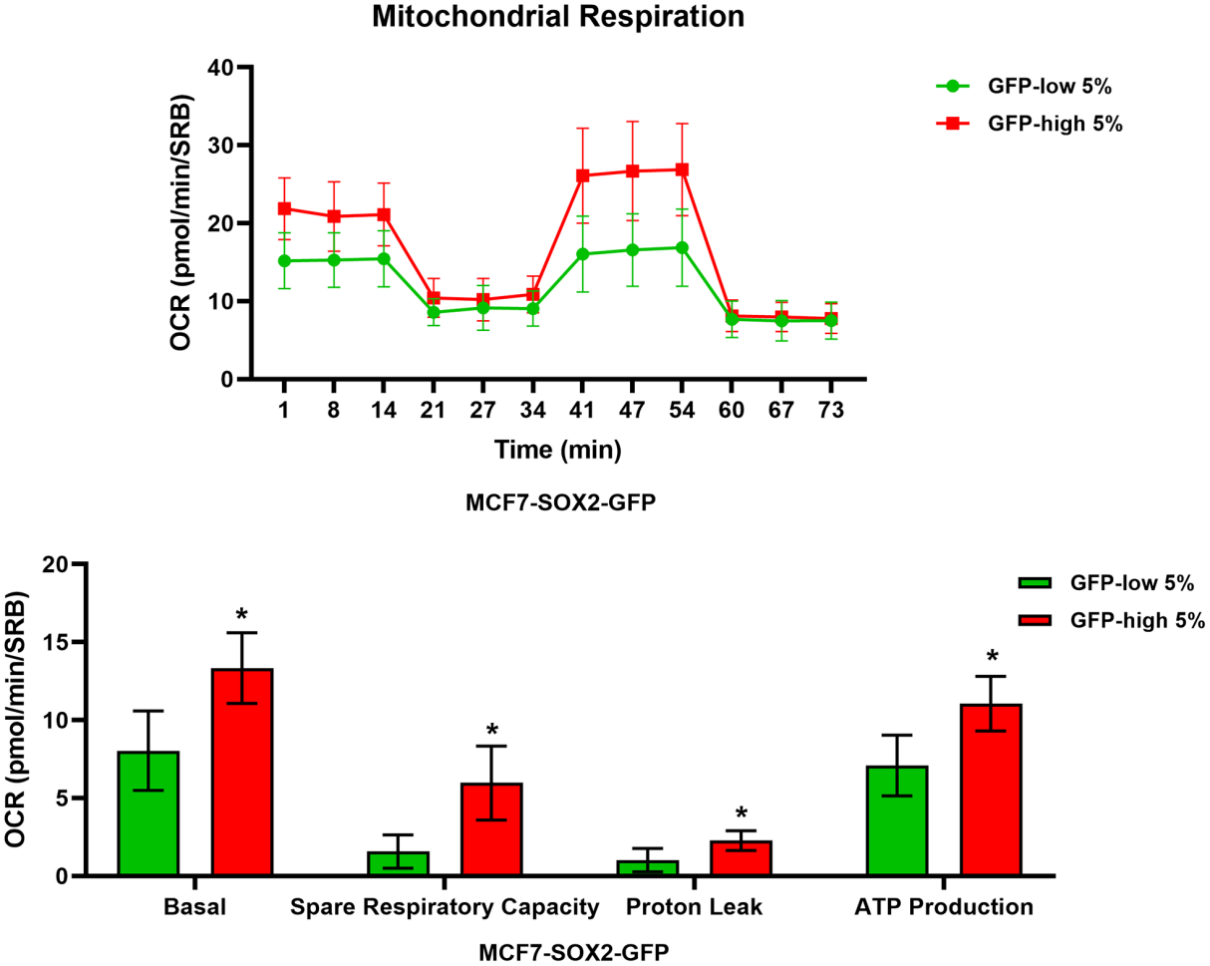
**Mitochondrial respiration is significantly enhanced in SOX2-high MCF7 cells.** MCF7 cells stably-transduced with the SOX2-GFP reporter construct were subjected to FACS sorting to isolate the 5% highest GFP (GFP-high) and the 5% lowest GFP (GFP-low) subpopulations. The Seahorse XF96 analyser was employed to measure the mitochondrial function. In the top panel, a representative OCR tracing is shown. In the lower panel, bar graphs show the basal respiration rate, spare respiratory capacity, proton leak and ATP production, obtained from the OCR quantification. Experiments were performed at least 3 times independently. Results are shown as the mean ± SD: *t*-test, two tailed-test, *p < 0.05.

**Figure 4:**
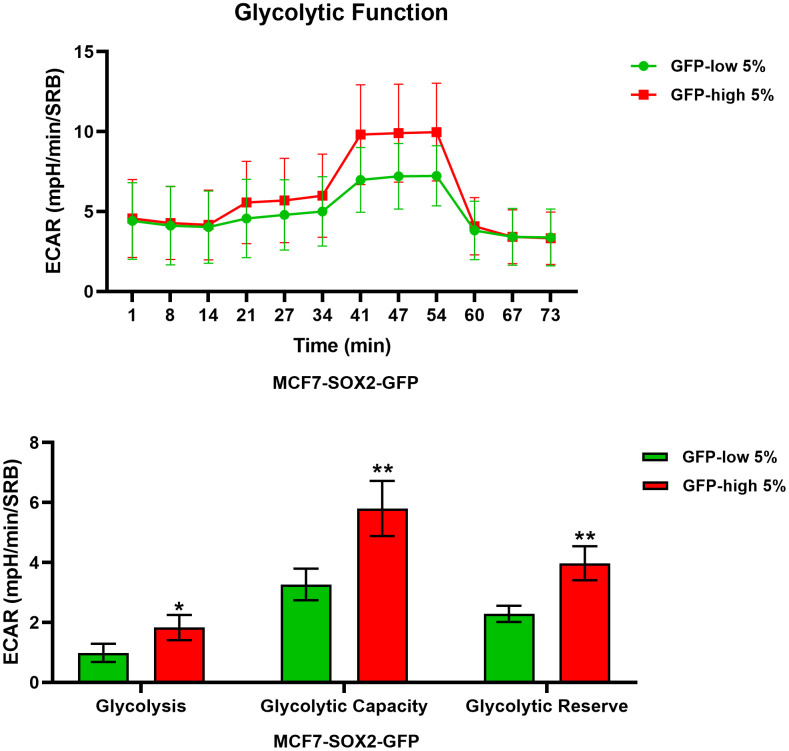
**Glycolysis is significantly enhanced in SOX2-high MCF7 cells.** MCF7 cells stably-transduced with the SOX2-GFP construct were subjected to FACS sorting to isolate the 5% highest GFP (GFP-high) and the 5% lowest GFP (GFP-low) subpopulations. The Seahorse XF96 analyser was employed to measure glycolytic function. In the top panel, a representative ECAR tracing is shown. In the lower panel, bar graphs show the glycolytic rate, glycolytic capacity and glycolytic reserve, obtained from the ECAR quantification. Experiments were performed at least 3 times independently. Results are shown as the mean ± SD: *t*-test, two tailed-test, *p < 0.05, ** p < 0.01.

We next analysed their metabolic flux activity, using the Seahorse XFe96 bioenergetic analyser. Figures 3, 4 show that the SOX2-high population is clearly more metabolically active than the SOX2-low cells, with significant increase in both oxygen consumption and glycolytic rates. This was consistent with the increase in mitochondrial mass, that we observed using MitoTracker Deep-Red.

**Figure 5:**
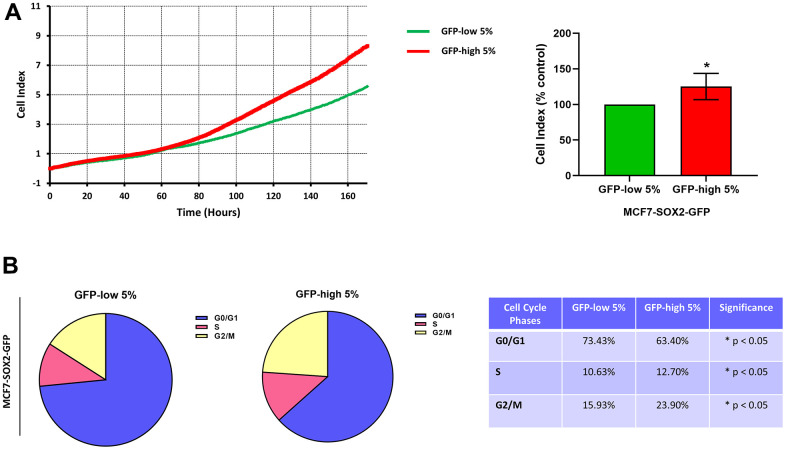
**Proliferation and cell cycle progression are elevated in the SOX2-high MCF7 cells.** MCF7 cells stably-transduced with the SOX2-GFP construct were subjected to FACS sorting to isolate the 5% highest GFP (GFP-high) and the 5% lowest GFP (GFP-low) subpopulations. (**A**) Proliferation was assessed using the xCELLigence RTCA system. In the left panel, a representative tracing is shown. In the right panel, the graph shows the average of the final cell index of at least 3 independent experiments. (**B**) Cell cycle was evaluated with propidium iodide by flow cytometry. The percentage of cells in G0/G1, S, and G2/M phases of the cell cycle are also represented in a tabular format. Experiments were performed at least 3 times independently. Results are shown as the mean ± SD: *t*-test, two tailed-test, *p < 0.05.

Finally, the SOX2-high cells were more proliferative than the SOX2-low cells (Figure 5A). Similarly, we observed by cell cycle analysis (Figure 5B) that SOX2-high cells showed a significant decrease in the G0/G1-phase, with corresponding increases in S-phase and G2/M-phase.

### SOX2-high MCF7 cells show increased drug resistance to Tamoxifen

**Figure 6:**
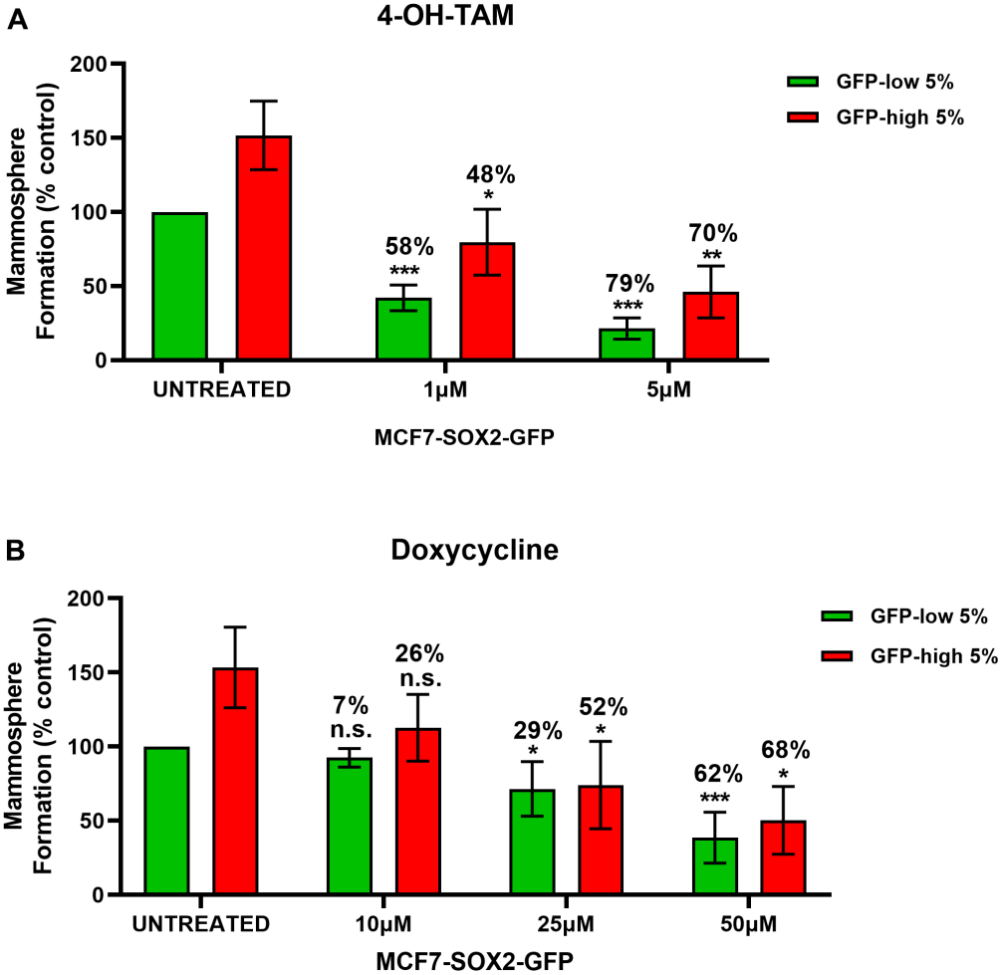
**SOX2-high MCF7 show resistance to treatment with 4-OH-Tamoxifen, but show higher sensitivity to Doxycycline.** MCF7 cells stably-transduced with the SOX2-GFP construct were subjected to FACS sorting to isolate the 5% highest GFP (GFP-high) and the 5% lowest GFP (GFP-low) subpopulations. Differential sensitivity of GFP-high and GFP-low subpopulations to (**A**) 4-OH-Tamoxifen and (**B**) Doxycycline at the indicated concentrations was evaluated by a mammosphere assay. The GFP-high and GFP-low subpopulations were plated in low-attachment plates for mammosphere assays and incubated with 4-OH-Tamoxifen or Doxycycline at the indicated concentrations. The number of mammospheres were analysed after 5 days. The percentage at the top of the bars represent the decrease of that bar compared with its own untreated control (GFP-high treated compare with GPF-high untreated; and GFP-low treated compared with GFP-low untreated). Experiments were performed at least 3 times independently. Results are shown as the mean ± SD: *t*-test, two tailed-test, *p < 0.05, ** p < 0.01, ***p < 0.001, n.s. not statistically significant.

In Figure 6A, we examined the differential sensitivity of SOX2-high and SOX2-low MCF7 cells sub-populations to 4-OH-Tamoxifen, an FDA-approved drug used in ER (+) breast cancer cells treatment. Interestingly, mammosphere formation by SOX2-low cells was more sensitive to Tamoxifen treatment, resulting in a reduction by 58% at 1μM and by 79% at 5μM, as compared with vehicle-treated control SOX2-low cells. However, mammosphere formation by SOX2-high cells show less decrease (~ 10%) as mammosphere formation is reduced by 48% at 1μM and by 70% at 5μM, as compared with vehicle-treated control SOX2-high cells. These results indicate that SOX2-high cells are more Tamoxifen-resistant than SOX2-low cells.

### SOX2-high MCF7 cells show more sensitivity to Doxycycline

We also evaluated the effect of Doxycycline (Figure 6B), an FDA-approved antibiotic which inhibits mitochondrial biogenesis [10], on the mammosphere formation of SOX2-high and SOX2-low MCF7 cell sub-populations. Note that SOX2-high cells were more sensitive to Doxycycline treatment, resulting in a reduction by 26% at 10μM, by 52% at 25μM and by 68% at 50μM, as compared with vehicle-treated control SOX2-high cells. SOX2-low cells show a mammosphere reduction by 7% at 10μM, by 29% at 25μM and by 62% at 50μM, as compared with vehicle-treated control SOX2-low cells. Therefore, SOX2-high cells were more effectively targeted by Doxycycline.

### SOX2-high MDA-MB-231 cells show increases in cell migration and invasiveness

**Figure 7:**
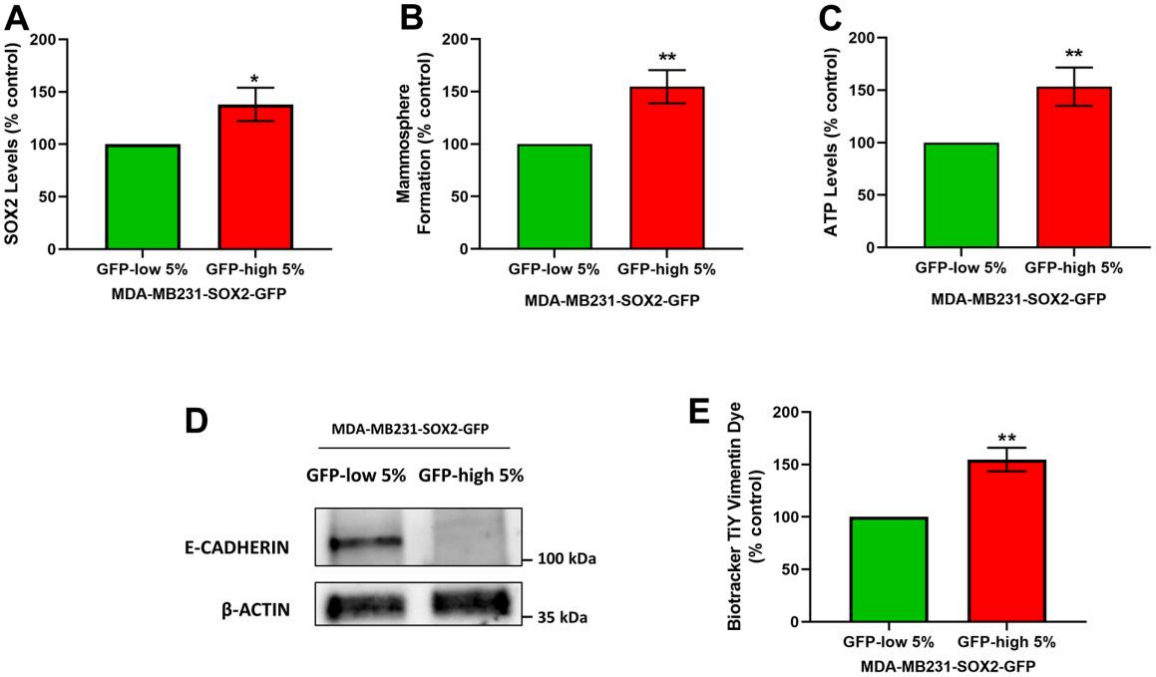
**SOX2-high MDA-MB-231 cells form mammospheres with greater efficiency, produce more ATP, and show a loss of E-cadherin expression.** MDA-MB-231 cells stably-transduced with the SOX2-GFP construct were subjected to FACS sorting to isolate the 5% highest GFP (GFP-high) and the 5% lowest GFP (GFP-low) subpopulations. (**A**) SOX2 levels of GFP-high and GFP-low subpopulations were quantitated by flow cytometry. (**B**) The GFP-high and GFP-low subpopulations were seeded in low-attachment plates for mammosphere assays and analysed after 5 days. (**C**) The GFP-high and GFP-low subpopulations were plated in complete DMEM medium and incubated with the Cell-Titer-Glo 2.0 Reagent for 15 min to determine the ATP levels. Also, two EMT markers were analysed. More specifically, (**D**) E-cadherin was analysed by Western blotting. β-actin was used as a protein loading control. Independently, (**E**) Vimentin levels were determined with the Biotracker TiY Vimentin Dye, by flow cytometry. Experiments were performed at least 3 times independently. Results are shown as the mean ± SD: *t*-test, two tailed-test, *p < 0.05, ** p < 0.01.

It is known that CSCs are responsible for tumour recurrence and metastatic spread, via cell migration and invasion. Since the SOX2-high show a stemness phenotype, we also checked their migration and invasion phenotypes. For that purpose, we used MDA-MB-231 cells as they are a well-established model for the study of cell motility and metastasis. Using the same protocol that was followed for the MCF7 cells to separate the two subpopulations, we obtained SOX2-high MDA-MB-231 cells and SOX2-low MDA-MB-231 cells. As a first step, we examined the SOX2 levels (Figure 7A), mammosphere formation (Figure 7B) and the ATP levels (Figure 7C) to validate that they showed the same phenotypic characteristics as MCF7 cells. Similarly, SOX2-high MDA-MB-231 cells showed increased SOX2 levels as measured by flow cytometry, as well as mammosphere forming ability, showing enhanced anchorage-independent growth, and characteristically higher ATP levels, than SOX2-low MDA-MB-231 cells.

We next evaluated the status of two known markers of the epithelial mesenchymal transition (EMT), namely E-cadherin and Vimentin. E-cadherin levels were assessed by Western blot analysis, and Vimentin levels were quantitated by FACS analysis, using Biotracker TiY Vimentin Dye. It has been reported this fluorescent probe permits the Vimentin identification and facilitates the visualization and enrichment of active tumour initiating cells [11]. Interestingly, we observed a decrease in the E-cadherin protein levels (Figure 7D) and an increase in the Vimentin levels in SOX2-high MDA-MB-231 cells (Figure 7E), confirming EMT activation.

**Figure 8:**
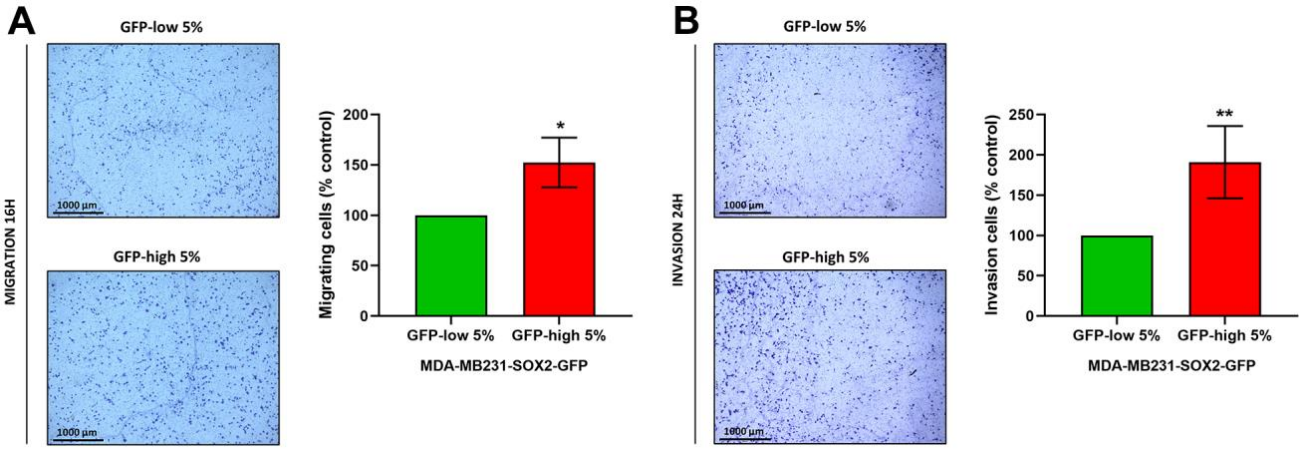
**Cell migration and cell invasion capacity are increased in SOX2-high MDA-MB-231 cells.** MDA-MB-231 cells stably-transduced with the SOX2-GFP construct were subjected to FACS sorting to isolate the 5% highest GFP (GFP-high) and the 5% lowest GFP (GFP-low) subpopulations. (**A**) The migratory capacity of the two subpopulations was assessed using Transwell-24 wells with uncoated PET membranes. The cells were allowed to migrate across an 8-μm pore uncoated membrane for 16 hours. In the left panel, the photos show the migration for a representative experiment. In the right panel, the bar graph shows the quantification of the migration events. (**B**) The invasion capacity of the two subpopulations was assessed using Transwell-24 wells, with pre-coated extracellular matrix proteins (PET membrane). The cells were allowed to pass across an 8-μm pore pre-coated membrane for 24 hours. In the left panel, the photos show the invasion for a representative experiment. In the right panel, the bar graph shows the quantification of the migration. Experiments were performed at least 3 times independently. Results are shown as the mean ± SD: *t*-test, two tailed-test, *p < 0.05, ** p < 0.01.

Finally, migration and invasion experiments performed with Transwells directly demonstrated that SOX2-high MDA-MB-231 cells indeed were more migratory (Figure 8A) and invasive (Figure 8B), since the number of cells that had crossed the membrane were greater. Taken together, these results show that SOX2-high MDA-MB-231 cells show a more aggressive, motile and invasive phenotype, consistent with increased stemness.

## DISCUSSION

Given that cancer stem cells (CSCs) are considered to be responsible for cancer relapse, therapy-resistance and metastatic dissemination, eliminating CSCs would be expected to prevent cancer recurrence and improve patient outcome. Thus, therapeutic targeting of CSCs could be an important new strategy for achieving more effective cancer therapy and long-term survival [12]. As a result, the detection, isolation, and characterization of CSCs are essential to understand tumour cell behaviour and develop new therapies.

Since CSCs only represent a sub-fraction of the total tumour mass and they share similar transcription and signalling pathways with normal stem cells, their identification and isolation has been quite challenging. Currently, different methods can be used to identify and isolate CSCs, such as magnetic-activated cell sorting (MACS) using magnetic microbeads linked to a specific monoclonal antibody or fluorescent-activated cell sorting (FACS) using fluorescence staining, mostly based on specific cell surface biomarkers. Although they are reliable methods, more functional and mechanistic models are necessary [3].

CSCs are regulated by several important transcription factors, so here we present a new approach to isolate and characterize the CSCs, using an enhancer recognition element of one of these transcriptional factors, linked to a GFP-based reporter system. This allowed us to isolate two distinct sub-populations of cells, according to the levels of GFP fluorescence. GFP-high cells would correspond to high transcriptional factor activity and levels and, we predicted that, this sub-population would be enriched CSCs. In contrast, GFP-low cells would correspond to a non-stem-like sub-population, which would serve as an internal control and comparative reference point.

More specifically, we selected SOX2, NANOG and TCF/LEF1, three transcription factors previously implicated in the characteristics of CSCs, to potentially isolate more stem-like cell sub-populations. These transcription factors have been widely-reported to be necessary for the maintenance of the normal and cancer stem cell features.

In breast cancer, Tamoxifen-resistant MCF7 cells express higher levels of SOX2 than parental MCF7 cells. Also, SOX2 over-expression increases the proportion of breast cancer stem cells by activating the Wnt signalling pathway [13]. The specific down-regulation of SOX2 by actinomycin D leads to depletion of CSCs, blocking the tumour-initiating capacity of breast cancer stem cells [14]. Moreover, Gong et al. identified the transcriptional repressor GATA binding 1 as a suppressor of SOX2 expression and, thereby, suppresses cancer stemness and tumourigenesis [15]. But the important role of SOX2 in CSC functions has not only been reported in breast cancer; silencing SOX2 in glioblastoma tumour-initiating cells drastically decreases their proliferative and tumourigenic potential [16], and SOX2 controls tumour initiation in squamous cell carcinoma [17]. On the other hand, over-expression of NANOG in colorectal CSCs promotes colony formation and tumourigenicity *in vivo* [18], and NANOG-shRNA transduced cancer cells exhibit decreased clonogenic growth and reduced proliferation [6]. Finally, LEF1 is an essential factor for stem cell maintenance and organ development, as its inhibition by Niclosamide attenuates cancer stemness and therapy resistance in colorectal cancer [19].

Here, we showed that SOX2-high MCF7 cells have a significant increase in their mammosphere forming ability, as well as in ATP production, indicating that this sub-population is functionally enriched in CSC activity. NANOG-high MCF7 cells and TCF/LEF1-high MCF7 cells also showed greater mammosphere forming ability and ATP production. However, the differences compared with GFP-low cells were not as significant. As such, we chose to focus on SOX2, as the reporter of choice for isolating the CSC sub-population.

We further validated this approach, by analysing different CSCs features. Firstly, we demonstrated by two methods, immunoblot analysis and flow cytometry, that the high transcriptional levels of SOX2 activity corresponded to the higher expression of the SOX2 protein. Secondly, we assessed the levels of CD44 and OCT4, well-known CSCs markers that are widely used to identify CSCs [2, 20]. SOX2-high MCF7 cells showed a significant increase of the CD44 and OCT4 levels.

Similarly, metabolic flux analysis was conducted using the Seahorse XFe96 to quantitatively measure oxidative mitochondrial and glycolytic activity. We observed a significant increase in both mitochondrial function and glycolysis in SOX2-high MCF7 cells. Furthermore, SOX2-high MCF7 cells were more proliferative, showing increases in the S-phase and the G2/M-phase of the cell cycle, confirming the presence of a proliferative and energetic CSC phenotype, with increased mitochondrial ATP production [1].

Drug resistance is another characteristic that helps to define the phenotype of CSCs. Chemo- and radio-resistance in CSCs has been reported in several studies [21]. In accordance with these previous observations, SOX2-high MCF7 cells also demonstrated drug resistance to Tamoxifen, an FDA-approved drug used in ER (+) breast cancer cells treatments. In many cases, Tamoxifen therapy clinically fails, due to an existing or by developing a resistant sub-population of cancer cells, that are the mechanistic drivers of tumour recurrence and metastasis. Doxycycline, an FDA-approved antibiotic which inhibit mitochondrial biogenesis, can be used to eradicate cancer stem cells as several studies have shown [10, 22]. Our results indicate that SOX2-high MCF7 cells show more Doxy-sensitivity, due to their higher dependence on mitochondrial metabolism, as compared with SOX2-low MCF7 cells. Mitochondrial biogenesis inhibition by Doxycycline produces a loss of SOX2-high MCF7 cells with the phenotypic characteristic of Tamoxifen-resistance.

CSCs have also been implicated in cell motility and invasiveness, resulting clinically in metastatic dissemination. To study this process further, we used MDA-MB-231 cells, a well-established model for the study of cell motility, invasion, and metastasis. SOX2-high MDA-MB-231 cells have higher expression levels of SOX2 protein, as compared with SOX2-low MDA-MB-231 cells, directly validating the model. Remarkably, SOX2-high MDA-MB-231 cells showed increases in their capacity to undergo both cell migration and invasion. To further validate this phenotype, we examined the expression of EMT markers in these cell populations, as EMT activation is necessary for the dissemination of the cancer cells to distant tissues [23]. Also, we examined their ability to undergo anchorage-independent growth (mammosphere assay) and their energetic status (ATP levels), confirming that SOX2-high MDA-MB 231 cells possess a stem-like and hyper-energetic phenotype.

In summary, our current work has provided a novel strategy to enrich for a sub-population of more energetically active, hyper-proliferative and invasive cancer cells, based on the functional activation a self-renewal pathway in these cells. Thus, the high levels of activation of the transcription factor SOX2 allowed the purification of a sub-population enriched in CSCs, by combining a SOX2-driven GFP reporter with FACS sorting.

## MATERIALS AND METHODS

### Cell models and other reagents

MCF7 and MDA-MB-231 cells were obtained commercially from the American Type Culture Collection (ATCC). Cells were maintained in Dulbecco’s Modified Eagle Medium (DMEM; Sigma-Aldrich, #D6546) supplemented with 10% Fetal Bovine Serum (HI FBS; Gibco, #10082-147), 2mM Glutamax (Gibco, #35050-061), and 1% Penicillin-Streptomycin (Sigma-Aldrich, #P0781). Cells were grown at 37° C in a 5% CO_2_ humidified incubator. 4-OH-Tamoxifen and doxycycline monohydrate were purchased from Sigma-Aldrich (#D1822, #579002).

### Viral transduction and cell selection

Lentiviral constructs (System Biosciences; pGreenFire1™-Sox2-SRR2-EF1-Puro, #SR20071-PA-P; pGreenFire1™-TCF/LEF1-EF1-Puro, #TR013PA-P; pGreenFire1™-Nanog-EF1-Puro, #TR019PA-P) were amplified and used to stably-transduce MCF7 and MDA-MB-231 cells, with the LentiSuite™ Basic Kit (System Bioscences, #LV340A-1), according to manufacturer’s protocol. After cell transduction, the cell lines were selected with puromycin for two weeks.

MCF7 and MDA-MB-231 cells stably transduced with the different constructs were sorted using the SONY SH800 Cell Sorter, and the 5% highest (GFP-high) and the 5% lowest (GFP-low) sub-populations of cells were isolated. After sorting, these cell sub-populations were subjected to different functional assays, to characterize their phenotypic differences experimentally.

### Mammosphere formation efficiency (MFE)

After sorting, the single cell suspensions were plated at a density of 500 cells/cm2 in mammosphere medium (DMEM F12, Gibco, #21041-025; B27, Gibco, #17504-044; 20ng/ml EGF, Peprotech, #AF-100-15; 1% PenStrep) under non-adherent conditions, in tissue culture dishes that were previously coated with Poly 2-hydroxyethyl methacrylate (poly-HEMA, Sigma-Aldrich, #P3932). Cells were incubated in a humidified atmosphere at 37° C with 5% CO_2_ for 5 days. After incubation, mammospheres greater than 50μm were counted using a graticule.

### ATP assay using Cell-Titer-Glo 2.0

Cell Titer Glo 2.0 was obtained from Promega (#G9242). Ten thousand cells were seeded after sorting in a 96 well plate in complete DMEM medium and incubated with the Cell-Titer-Glo 2.0 Reagent in a humidified atmosphere at 37° C and 5% CO2 for 15 min. Luminescence content was evaluated using the Varioskan ™ LUX plate reader (ThermoFisher Scientific).

### Western blotting

After sorting, cells were washed with PBS (Gibco, #10010-023) and lysed in RIPA lysis buffer (Sigma-Aldrich, #R0278) containing protease and phosphatase inhibitors (Roche, #04906845001 #05892970001). Then, the cell lysates were centrifugated for 10 min at 13,000 rpm, and the supernatants were collected. Protein content was measured with the BCA protein assay kit (Thermo Scientific, #23225), before adding Laemmli sample buffer (Invitrogen, # NP0007) and boiled for 10 min at 99° C. Then, total cell lysates were loaded onto SDS-polyacrylamide gels (Mini-Protean TGX Gel, 4-20%, Bio-Rad, # 4561094). The gels were transfected to 0.2 μm nitrocellulose membranes (Mini Trans-Blot Turbo Transfer Pack, Bio-Rad, #1704158), using the TransBlot Turbo Transfer System (Bio-Rad). After transfer, membranes were blocked with 5% BSA PBS-T (PBS 1%; 0.5% Tween 20, Sigma-Aldrich, #) for 1 hour at room temperature in an orbital shaker. The membranes were subsequently incubated with primary antibodies in 5% BSA PBS-T for 12–16 h at 4° C, followed by incubation with secondary antibodies (Cell Signalling Technology; Anti-Rabbit IgG HRP Linked #7074; Anti-Mouse IgG Linked #7076) in 5% BSA PBS-T for 1 h at room temperature. The membranes were developed with Supersignal West Pico chemiluminescent substrate (Thermo Scientific, #34580). Antibodies against the following proteins were used: SOX2 (Abcam, #ab93689), E-cadherin (Santa Cruz Biotechnology, #sc-8426), β-actin (Sigma-Aldrich, #A2228). β-actin was used as control for equal protein loading. The resulting images were acquired using GeneSys Software (Syngene).

### SOX2 analysis by flow cytometry

After FACS sorting, cells were fixed with ethanol 70% and incubated with SOX2 antibody (Abcam, #ab93689) and Alexa Fluor 660 anti-rabbit for 30 min at RT. Cells were washed and resuspended in 1% BSA PBS, and analysed by FACS (Attune ™ NxT Flow Cytometer, ThermoFisher Scientific).

### Mitochondrial staining

After FACS sorting, cells were stained with MitoTracker Deep Red (Invitrogen, #M22426) for 30 minutes at 37° C. Cells were washed and resuspended in PBS, and analysed by FACS (Attune ™ NxT Flow Cytometer, ThermoFisher Scientific).

### CD44 analysis

After FACS sorting, cells were incubated with CD44 antibody (APC mouse anti-Human CD44, BD Pharmingen. #559942) for 30 min on ice. Cells were washed and resuspended in 1% BSA PBS, and analysed by FACS (Attune ™ NxT Flow Cytometer, ThermoFisher Scientific).

### OCT4 analysis

After FACS sorting, cells were fixed with PFA 4%, permeabilizated with Triton 0.1% and incubated with OCT4-PE antibody (PE anti-OCT4, Sony. #3868520) for 1 h 30 min at RT. Cells were washed and resuspended in PBS, and analysed by FACS (Attune ™ NxT Flow Cytometer, ThermoFisher Scientific).

### Seahorse XFe-96 metabolic flux analysis

Real time oxygen consumption rates (OCRs) and Extracellular acidification rates (ECARs) were determined using the Seahorse Extracellular Flux (XFe96) analyser (Seahorse Bioscience). Fifteen thousand cells were plated in the XFe-96-well plates after sorting and incubated in a humidified atmosphere at 37° C with 5% CO_2_ for 24 hours. Then, cells were incubated in 175μl/well of XF assay media at 37° C, in non-CO_2_ incubator for 1 hour. During the incubation time, 25μl of 80 mM glucose, 9 μM oligomycin and 1M 2-deoxyglucose (for ECAR measurement) or 10 μM oligomycin, 10 μM FCCP, 10 μM rotenone and 10 μM antimycin A (for OCR measurement) in XF assay media was loaded into the injection ports of the XFe-96 sensor cartridge. Measurements were normalized by protein content (Sulforhodamine B assay). Data sets were analysed by XFe-96 software.

### xCELLigence RTCA system (Agilent)

Ten thousand cells were seeded after sorting into RTCA E-Plates (Agilent, #300601140) for measure the proliferation using RTCA (Real-Time Cell Analysis), using cell-induced electrical impedance.

### Cell cycle analysis

After FACS sorting, cells were incubated with propidium iodide, following the manufacturer’s recommendations (Muse® Cell Cycle Kit, Luminex, #MCH100106) and analysed by FACS (Attune ™ NxT Flow Cytometer, ThermoFisher Scientific).

### Analysis using the Biotracker TiY Vimentin Dye

After FACS sorting, cells were incubated with Biotracker TiY Vimentin Dye (Sigma-Aldrich. #SCT059) for 1 hour at 37° C in an incubator. Then, the cells were washed and resuspended in PBS, and analysed by FACS (Attune ™ NxT Flow Cytometer, ThermoFisher Scientific).

### Cell migration and invasion assays

Transwell-24 wells, with uncoated 8μm pores transparent PET membrane (Corning, #353097), were used for cell migration experiments, and Transwell-24 wells pre-coated with extracellular matrix proteins and 8μm pores PET membrane (Corning, #354480) were used for the cell invasion experiments.

In both types of experiments, fifteen thousand cells (migration) and twenty thousand cells (invasion) were plated after sorting into the upper chamber of the Transwell in a serum-free DMEM with 1% Penicillin-Streptomycin. The lower chamber was filled with complete culture medium (DMEM with 10% FBS, 2mM Glutamax, and 1% Penicillin-Streptomycin) as the chemo-attractant. Cells were incubated in a humidified atmosphere at 37° C and 5% CO_2_ for 16 hours in the migration experiments and 24 hours in the invasion experiments. Cells were removed from the upper surface by scrubbing with cotton swabs. Chambers were stained in 0.5% crystal violet for 15 minutes, rinsed in water and examined under a bright-field microscope. Values were obtained by counting four fields per membrane (20x objective) and represent the average of at least 3 independent experiments.

### Statistical analysis

Statistical significance was determined using the Student’s t-test; values of less than 0.05 were considered significant. Data are shown as the mean ± SD. All experiments were performed at least three times independently, with three or more technical replicates for each condition.
